# Wavelet Transform-Based UV Spectroscopy for Pharmaceutical Analysis

**DOI:** 10.3389/fchem.2018.00503

**Published:** 2018-10-26

**Authors:** Erdal Dinç, Zehra Yazan

**Affiliations:** ^1^Department of Analytical Chemistry, Faculty of Pharmacy, Ankara University, Ankara, Turkey; ^2^Department of Chemistry, Ankara University Faculty of Science, Ankara, Turkey

**Keywords:** discrete wavelet transform, continuous wavelet transform, fractional wavelet transform, UV spectroscopy, pharmaceutical analysis

## Abstract

In research and development laboratories, chemical or pharmaceutical analysis has been carried out by evaluating sample signals obtained from instruments. However, the qualitative and quantitative determination based on raw signals may not be always possible due to sample complexity. In such cases, there is a need for powerful signal processing methodologies that can effectively process raw signals to get correct results. Wavelet transform is one of the most indispensable and popular signal processing methods currently used for noise removal, background correction, differentiation, data smoothing and filtering, data compression and separation of overlapping signals etc. This review article describes the theoretical aspects of wavelet transform (i.e., discrete, continuous and fractional) and its characteristic applications in UV spectroscopic analysis of pharmaceuticals.

## Introduction

In experimental studies, instruments or devices can provide signals (or graphs) in different formats e.g., spectrum, chromatogram, voltammogram, and electroferogram etc. The analysis of chemicals and pharmaceuticals in various samples is based upon the utilization of the measured signals of substances of interest. In practice, such an analysis for a multicomponent mixture may not be determined without a prior separation step due to spectral overlapping. Therefore, high performance liquid chromatography (HPLC) is one of the most commonly used techniques for quantitative estimation in the quality control of raw materials and commercial products in laboratories. In some cases, chromatographic determination could not be possible due to not only similar physicochemical behavior of analytes but also time- and solvent-consumption for optimal experimental conditions.

In practice, UV spectroscopic methods are widely used in chemical and pharmaceutical analysis. As compared to chromatographic ones, the use of spectroscopic methods provides a rapid analysis with low-cost and acceptable results. However, multicomponent analysis may not be possible with a traditional UV spectrophotometric approach due to spectral interferences of both active and inactive ingredients in samples. In some cases, derivative spectrophotometry (O'Haver and Green, 1976; O'Haver, 1979; Levillain and Fompeydie, 1986; Ragno et al., 2006) and its improved versions e.g., ratio spectra-derivative spectrophotometry (Salinas et al., 1990), ratio spectra-derivative spectrophotometry-zero crossing (Berzas Nevado et al., 1992; Dinç and Onur, 1998; Dinç, 1999), and double-divisior-ratio spectra-derivative spectrophotometry (Dinç and Onur, 1998; Dinç, 1999; Gohel et al., 2014; Shokry et al., 2014) could be used in place of conventional UV spectrophotometric method for analysis of binary and ternary mixtures without using a separation step. However, these spectral approaches may not always yield successful data due to severely overlapping spectral bands, spectral noise and baseline variation. Additionally, high-order differentiation of spectra may lead to spectral deterioration i.e., a decrease in signal intensity and signal-to-noise ratio. As a result, a number of mathematical manipulations (or signal processing methods) are often required to make instrumental signals more meaningful for analysis purpose.

Generally speaking, transform (i.e., Fourier, Hilbert, short-time Fourier, Wigner distribution, Radon, and wavelet) is a very suitable technique in the pre-treatment step to simplify signals. Fourier transform (FT) is the first method to modify chemical signal (Griffiths, 1977; Cooper, 1978; Griffiths and De Haseth, 1986; Ernst, 1989) with the mathematical essence such as filtering, convolution/deconvolution etc. FT analysis can localize signal in frequency domain very well, but not so much in time domain. In contrast, wavelet transform (WT) has the advantage of localizing signals both in time (position) and frequency (scale) domains, making it a preferable mathematical tool to replace FT in the study of the local property of a signal and the removal of the perturbation of measuring error in spectral analysis. Nowadays, WT is one of the most signal analysis algorithms commonly used in the different fields of chemistry and engineering, providing alternative ways or opportunities to resolve complex spectral bands or diverse data types of signals.

For readers interested in learning the general theory of wavelets, more details can be found in the literature (Mallat, 1988; Chui, 1992; Daubechies, 1992; Newland, 1993; Byrnes et al., 1994; Chui et al., 1994; Vetterli and Kovačević, 1995; Strang and Nguyen, 1996).

In the signal smoothing and de-noising of spectral peaks, the elimination of noise requires an application of appropriate filters to the raw spectral data such as some conventional signal filters Savitzky–Golay, Fourier and Kalman (Brown et al., 1994, 1996). The use of WT in signal analysis is two-fold: (i) to detect the singularities of a signal very likely caused by high-frequency noise and (ii) to separate the signal frequencies at different scales (Palavajjhala et al., 1994; Yan-Fang, 2013; Li and Chen, 2014). To illustrate this, Barclay et al. (1997) performed a comparative study in de-noising and smoothing of Gaussian peak by using wavelet, Fourier and Savitzky–Golay filters i.e., smoothing eliminates high-frequency components of the transformed signal irrespective of their amplitudes, while de-noising eliminates small-amplitude components of the transformed signal irrespective of their frequencies.

Historically, WT principal applications in chemistry were first explored by Walczak and Massart (1997a), who presented an approach based on the application of wavelet packet transform (WPT) to the best-basis selection for the compression and de-noising of a set of signals in time-frequency domain. In their paper, the proposed technique was compared to Wickerhauser's approach (Wickerhauser, 1994) of fast approximate principal component analysis (PCA). These authors also published two more papers on the application of wavelets for data processing i.e., the introduction of WPT for noise suppression and signal compression (Walczak and Massart, 1997b) and the use of WT for signal compression and denoising, image processing, data compression and multivariate data modeling in analytical chemistry (Walczak and Massart, 1997c). On the other hand, Alsberg et al. (1997) tried to introduce WT to chemometricians by suggesting the short-time FT technique as a resolution to obtain information about frequency changes over time as well as the WT for de-noising, baseline removal, determination of derivative zero crossings and signal compression. In 1997, WT application in chemical analysis was also confirmed by Wang et al. (1997) and Depczynski et al. (1997). Up to date, WT processing of the different types of raw signals has been reported for liquid chromatography (Shao et al., 1997, 1998a,b,c) and NMR spectroscopy (Neue, 1996; Barache et al., 1997), Raman spectra (Cai et al., 2001; Ehrentreich and Summchen, 2001), and voltammetry (Chen et al., 1996; Fang and Chen, 1997; Zheng et al., 1998; Zhong et al., 1998; Aballe et al., 1999; Zheng and Mo, 1999) IR and Raman spectroscopy (Shao and Zhuang, 2004; Hwang et al., 2005; Chalus et al., 2007; Jun-fang et al., 2007; Lai et al., 2011). In this context, as in the various fields of mathematics and engineering, the implementations of WT in analytical chemistry and neighbor disciplines has become increasingly attractive as an alternative way to analyze complex mixtures previously unresolved by traditional analytical techniques.

With reference to the above-mentioned review, the aim of this paper is to describe the fundamentals of WT methodologies and its typical implementations for UV spectroscopic analysis of pharmaceuticals.

## Brief history of wavelets

In the literature, the first study was related to the Haar Wavelet transform. This family was suggested by the mathematician Alfred Haar in 1909. However, the word “wavelet” was not used in the period of Haar. In fact, the word “wavelet” was invented by Morlet and the physicist Alex Grossman in 1984. After the first orthogonal Haar wavelet, the second orthogonal wavelet known as “Meyer wavelet” was formulated by the mathematician Yves Meyer in 1985. In 1988, Stephane Mallat and Meyer elaborated the concept of multiresolution. In the same year, a systematical method to construct compactly supported continuous wavelets was found by Ingrid Daubechies. Afterwards, Mallat proposed the fast wavelet transform. The emergence of this algorithm increased the implementations of the WT in the signal processing field.

In other words, the history of the wavelet families could be given in the following chronological order: Haar families in 1910, Morlet wavelet concept in 1981, Morlet and Grossman, “wavelet” in 1984, Meyer, “orthogonal wavelet” in 1985, Mallat and Meyer, multiresolution analysis in 1988, Daubechies, compact support orthogonal wavelet in 1988 and Mallat, fast wavelet transform in 1989 (c.f. Chun-Lin, 2010).

Basically, WT can be mainly classified into discrete wavelet transform (DWT) and continuous wavelet transform (CWT) in the signal analysis. The theory and implementations of wavelets in chemistry and related fields were well documented as review papers (Leung et al., 1998; Dinç and Baleanu, 2007b; Dinç, 2013; Li and Chen, 2014; Medhat, 2015) and reference books (Walczak and Massart, 2000a,b; Walczak and Radomski, 2000; Brereton, 2003, 2008; Chau et al., 2004; Danzer, 2007; Mark and Workman, 2007; Dubrovkin, 2018).

## Wavelet transform algorithms

**Figure 1:**
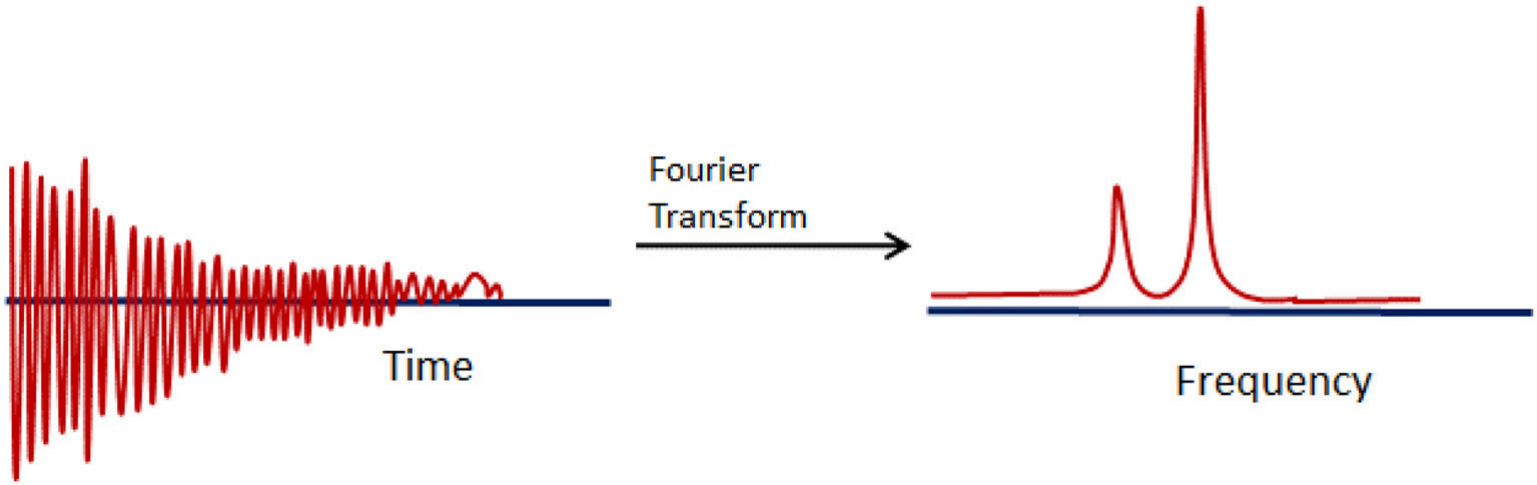
Representation of Fourier transform of a signal from time domain to frequency domain.

FT is based upon the decomposition of a signal into a set of trigonometric (sine and cosine) functions i.e., FT represents a signal in terms of sinusoids. The representation of FT of a signal from time mode to frequency mode is illustrated in Figure 1. For the determination of a local information in the FT, it is required to use an analyzing function ψ having localization properties in both frequency and time domains. This ψ function is named as a wavelet and it must be wave of finite duration.

WT contains the decomposition of a signal into a set of basic functions (wavelets). Basis functions of WT are small waves detected in different times. On the contrary to FT, WT gives information on both time and frequency, making it as an alternative approach to eliminate the resolution problem in signal analysis.

By definition, wavelets are the mathematical methods that convert the data into various coefficients and then analyze each coefficient at a resolution corresponding to its scale. Projection of a signal onto wavelet basic functions is called the wavelet transform. In other words, wavelets are mathematical functions generated from a mother wavelet Ψ(x) by the scaling parameter (dilatation) and shifting parameter (translation) i.e., the signal is expanded on a set of the dilatation (*scaling parameter*) of functions 

(1) $$\psi \left(\frac{x-a}{b}\right)$$

The scaling parameter has a significant role for the variation of time and frequency resolution when processing the signal.

For a given mother wavelet (Daubechies, 1992) ψ(*x*) by the scaling parameter and shifting parameter o fψ(*x*), a set of functions expressed by ψ_*a, b*_(*x*) is obtained from the following equation.

(2) $$\psi_{a,b\;}\left(x\right)=\;\frac{1}{\;\sqrt{\;\left|a\right|\;}\;}\;\psi \;\left(\frac{x-b}{a}\right),\text{a}\neq \text{0},\text{a},\text{b}\in \text{R}$$

where *a* is the scaling parameter, *b* is the shifting parameter and R is domain of real number. The mathematical expression of a CWT on a function *f* (x) is given below 

(3) $$CWT\left\{f\left(x\right);a,b\right\}=\int_{-\infty }^{\infty }f(x)\psi_{a,b}^{*}(x)dx=\langle f(x),\psi_{a,b}\rangle$$

here the superscript ^*^ is related to the complex conjugate and 〈*f*(*x*), ψ_*a, b*_〉 represents the inner product of function f(x) onto the wavelet function ψ_a, b_(x).

The original signal can be completely reconstructed by a sampled version of the CWT. Usually, the exemplar is follows as

(4) $$a=2^{-\text{m}}\text{and }b={\text{n2}}^{-\text{m}}$$

Here *a* and *b* denote scale and dilatation parameters, respectively, and *R* is the real number. The expression of DWT can be given as

(5) $$DWT=\;\int_{-\infty }^{+\infty }f\;\left(X\right)\;\psi_{m,\;n}^{*}\left(x\right)dt$$

Where $\psi_{\text{m},\;\text{n}}^{*}\left(X\right)=2^{-\text{m}}\;\psi \;\left(2^{\text{m}}\text{ x}-\text{n}\right)$ is the dilated and translated version of the mother wavelet. In the application of the DWT, only outputs from the low-pass filter are processed by WT. However, in the wavelet packet decomposition of signals, both outputs from the low-pass and high-pass filters are manipulated by WT (Strang and Nguyen, 1996). Multiresolution decomposition with wavelets is an interesting topic for signal and image analysis (Mallat, 1988; Daubechies, 1992).

Some families of wavelets with names and their coding list are illustrated in Table 1.

**Table 1:** Families of wavelets with names and their coding list.

**Wavelet families**	**Coding**
Haar	haar
Daubechies	db
Symlets	sym
Coiflets	coif
BiorSplines	bior
ReverseBior	rbio
Meyer	meyr
Dmeyer	dmey
Gaussian	gaus
Mexican hat function	mexh
Morlet	morl
Complex Gaussian	cgau
Shannon	shan
Frequency B-Spline	fbsp
Complex Morlet	cmor

For signal processing, there is also another WT approach i.e., fractional wavelet transform (FWT) specifically designed for rectification of the limitations of the WT and fractional FT (Blu and Unser, 2000, 2002; Unser and Blu, 2000). FWT is based on the fractional B-splines. As it is already known, the splines play an important role on the early development of the theory of WT.

A B-spline is generalization of the Beziers curve. Let a vector known as the knot be defined by *T* = {*t*_0_*, t*_1_*, …, t*_*m*_} where T is a non-decreasing sequence with *t*_*i*_ ϵ [0, 1], and define control point *P*_0_, *P*_*n*_. The knots *t*_0_*, t*_1_*, …, t*_*m*_ is called internal knots. If *p* = *m*- *n*- 1 denotes the degree, the basis function is defined as follows: 

(6) $$N_{i,\;0}(t)=f\left(x\right)=\;\{\begin{array}{c} 1,\;if\;t_{i}\leq t\;<t_{i+1}\;and\;t_{i+1}\\ 0\;otherwise \end{array}$$

and

(7) $$N_{i,\;p\;}\left(t\right)=\;\frac{\;t\;-\;t_{i}\;}{t_{i+p}\;-\;t_{i}\;}\;N_{i,\;p-1}\;\left(t\right)+\;\frac{\;t{\;}_{i+p+1}-\;t\;}{t_{i+p}+1\;-\;t_{i+1}\;}\;N_{i+1,\;p-1}\;\left(t\right)$$

Therefore, the curve defined by

(8) $$C\;(t)=\;\sum_{i=0}^{n}P_{i}\;N_{i,\;p}\;(t)$$

is a B-spline

Fractional B-spline: The fractional B-spline is defined as

(9) $$\beta_{+}^{\alpha }(x)=\frac{\;\sum_{k=0}^{+\infty }\;{\left(-1\right)}^{k}\;\left(\begin{array}{c} \alpha +1\\ k \end{array}\right){\left(x-k\right)}_{+}^{\alpha }\;}{\Gamma \;(\alpha +1)}$$

where Euler's Gamma function is obtained by

(10) $$\Gamma \;\left(\alpha +1\right)=\;\int_{0}^{+\alpha }x^{\alpha }\;e^{-x}\;dx$$

and

(11) $${\left(x-k\right)}_{\alpha }^{+}=\max{\left(x-k,\;0\right)}^{\alpha }$$

The forward fractional finite difference operator of order α is defined as

(12) $$\Delta_{+}^{\alpha }\;f\left(x\right)=\;\sum_{k=0}^{+\infty }{\left(-1\right)}^{k}\;{(}_{k}^{\alpha })\;f\;\left(x-k\right),$$

where 

(13) $$\left(\frac{\alpha }{k}\right)=\;\frac{\;\Gamma \;\left(\alpha +1\right)\;}{\;\Gamma \left(k+1\right)\left(\alpha -k+1\right)\;}$$

B-splines fulfill the convolution property, namely

(14) $$\beta_{+}^{\alpha 1}*\beta_{+}^{\alpha 2}=\;\beta_{+}^{\alpha 1+\alpha 2}$$

The centered fractional B-splines of degree α is defined as 

(15) $$\beta_{\alpha }^{*}\left(x\right)=\;\frac{\;1\;}{\;\Gamma \;\left(\alpha +1\right)}\;\sum_{k\in Z}^{\;}{\left(-1\right)}^{k}\;\left|\frac{\alpha +1}{k}\right|\;{\left|x-k\right|}_{\alpha }^{*}$$

where

(16) $${\left|x\right|}_{-}^{\alpha }=\;\{\begin{array}{c} \frac{\;{\left|\;x\right|}^{\alpha }\;}{-2\sin(\frac{\pi }{2}\alpha )},\;\alpha \;not\;even\\ \frac{X\log x\;}{\;{\left(-1\right)}^{1+n}\;\pi \;},\;\alpha \;even \end{array}$$

The fractional B-spline wavelet is defined as

(17) $$\begin{array}{l} \psi_{+}^{\alpha }\;(\frac{x}{2})\;=\overset{\;}{\underset{k\in Z}{\sum }}\frac{{\left(-1\right)}^{k}}{2^{\alpha }}\;\\ \;\overset{\;}{\underset{1\in Z}{\sum }}(\begin{array}{c} \alpha +1\\ 1 \end{array})\beta_{*}^{2\alpha +1}(1+k-1)\beta_{+}^{\alpha }\;(x-k) \end{array}$$

We mention that the fractional splines wavelets of degree obey the following

(18) $$\int_{-\infty }^{+\infty }X^{n}\;\psi_{+}^{\alpha }\;\left(x\right)dx=0,\;\ldots ,\;[\alpha ]$$

and the Fourier transform fulfills the following relations 

(19) $${\hat{\psi }}_{+}^{\alpha }\left(\varpi \right)=C\;{\left(j\varpi \right)}^{\alpha +1},\;as\;\varpi \to 0$$

and

(20) $${\hat{\psi }}_{\alpha }^{*}\left(\varpi \right)=C\;{\left(j\varpi \right)}^{\alpha +1},\;as\;\varpi \to 0$$

where ${\hat{\psi }}_{+}^{\alpha }\left(\varpi \right)$is symmetric. The fractional spline wavelet behaves like a fractional derivative operator.

## Strategies in CWT applications to UV spectroscopy analysis of multicomponent mixtures

For the past 15 years, the potential application of CWT in chemistry, especially in combination with other mathematical methods, leads us to a conclusion that WT has interestingly became a useful algorithm for UV quantitative analysis of pharmaceuticals. Four different models [i.e., continuous wavelet transform-zero crossing (CWT-ZC), ratio spectra-continuous wavelet transform (RS-CWT), ratio spectra-continuous wavelet transform-zero crossing (RS-CWT-ZC), and double divisor ratio spectra-continuous wavelet transform (DDRS-CWT)] were described in the implementation of CWT to UV spectroscopic data for the resolution of overlapping spectra to quantify drugs in different types of samples. The modeling of CWT—UV spectroscopic approaches are detailed below. Fundamentally, these approached can be successfully applied to the UV spectroscopic analysis of binary and ternary mixtures, provided that the law of additivity of absorbance is obeyed.

## Continuous wavelet transform-zero crossing

The application of CWT-ZC approach to UV spectroscopic signals was first proposed by Dinç and Baleanu (2003a).

**Figure 2:**
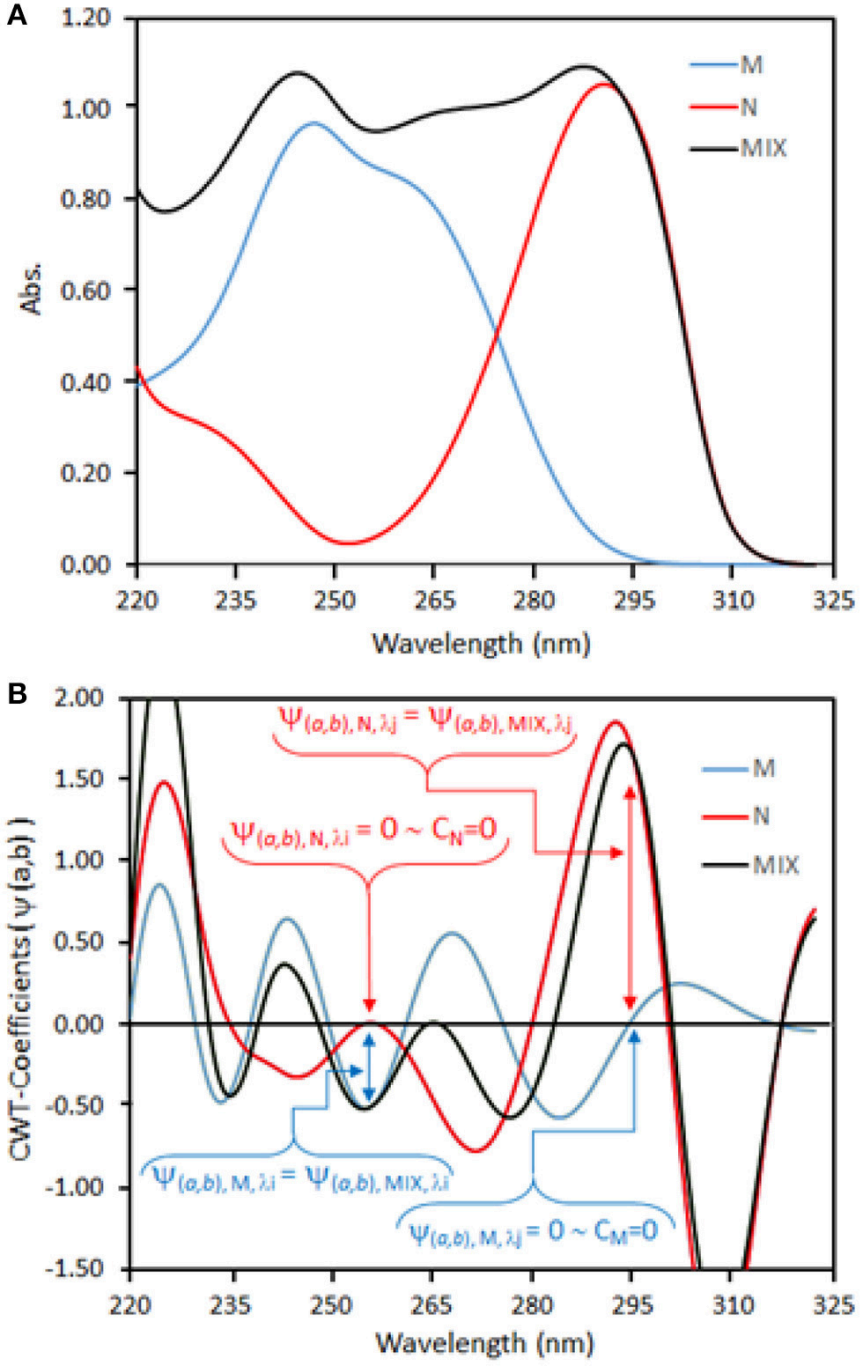
**(A)** Absorption spectra and **(B)** CWT spectra of M (**–**) and N (**–**) compounds and their mixture (**–**).

If a mixture of two analytes (M and N) is considered (see Figure 2A) and the absorbance of this binary mixture is measured at λ_*i*_, we can have the following equation (Charlotte Grinter and Threlfall, 1992):

(21) $${\text{A}}_{mix,\;\lambda i}\;=\;\alpha_{\text{M},\text{ λi}}{\text{C}}_{\text{M}}+\;\beta_{\text{N},\text{ λ}i}{\text{C}}_{\text{N}}$$

where Am_λ*i*_ is the absorbance of the binary mixture at wavelength λ_*i*_, and the coefficients are the absorptivities of M and N, respectively. C_M_ and C_N_ represent the concentrations of M and N, respectively.

If CWT is applied to Equation (21), the following function can be obtained as

(22) $${\text{ψ}}_{\left(\text{a}.\text{b}\right),\text{ MIX},\;\lambda i}=\;{\text{ψ}}_{\left(\text{a}.\text{b}\right),\text{M},\;\lambda \text{i}}\;{\text{C}}_{\text{M}}\;+\;{\text{ ψ}}_{\left(\text{a}.\text{b}\right),\text{ N},\;\lambda i}\;{\text{C}}_{\text{N}}$$

If ψ_(a.b), N, λ*i*_C_N_ = 0, then we obtain the following equation

(23) $${\text{ψ}}_{\left(\text{a}.\text{b}\right),\text{ MIX},\;\lambda i}=\;{\text{ψ}}_{\left(\text{a}.\text{b}\right),\text{ M},\;\lambda i}\;{\text{C}}_{\text{M}}$$

Equation (23) shows that CWT (ψ_(*a*.*b*), *M*, λ*i*_ *C*_*M*_) amplitudes of M in the binary mixture are dependent only on C_M_ regardless of C_N_ (see Figure 2B).

## Ratio spectra-continuous wavelet transform

Apart from CWT-ZC approach, overlapping spectral bands in a binary mixture could be solved by the application of a combined hybrid approach i.e., RS-CWT (Dinç and Baleanu, 2004a,c).

**Figure 3:**
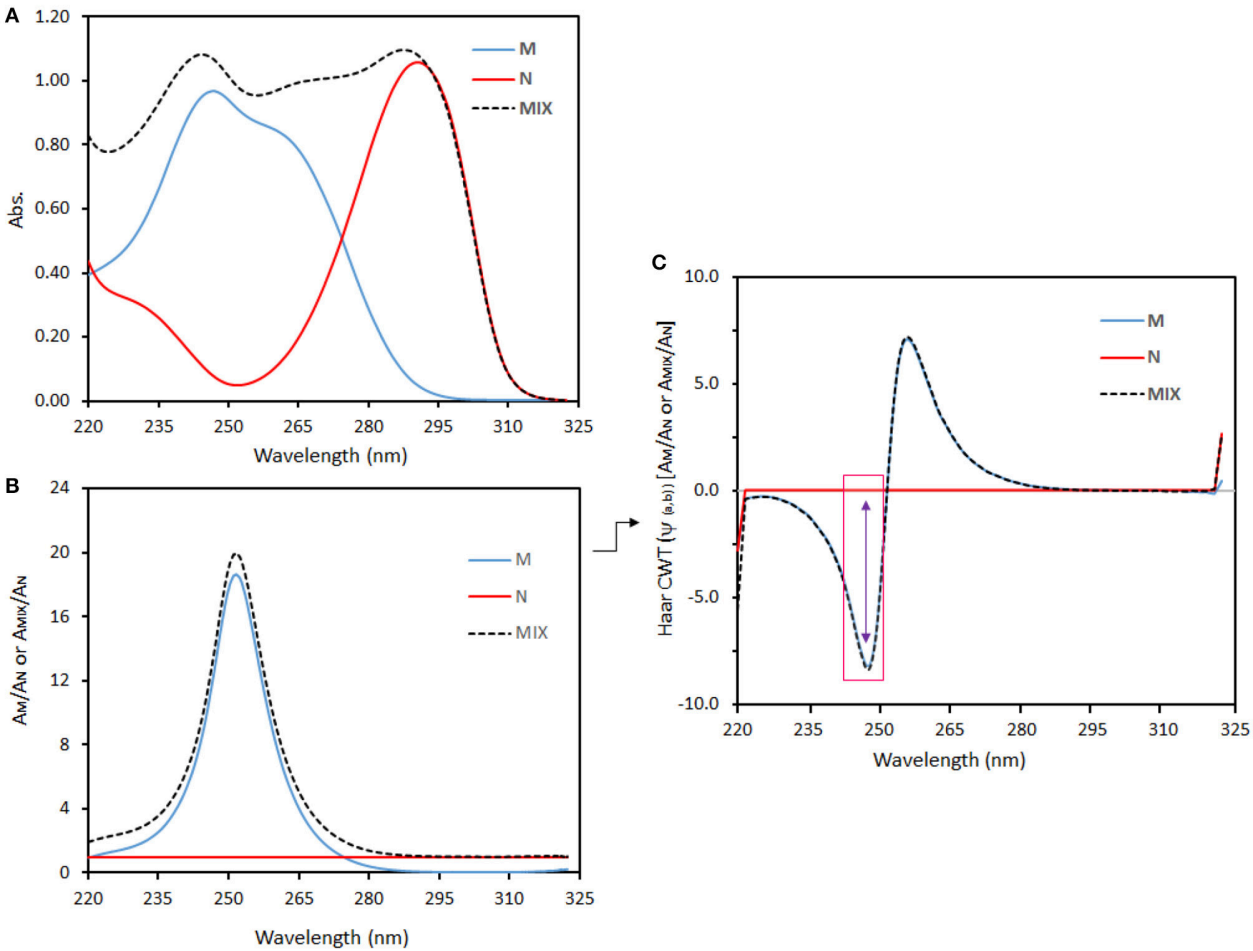
**(A)** Absorption spectra, **(B)** ratio spectra, and **(C)** Haar CWT spectra of M and N compounds and their binary mixture.

The absorption spectra of M and N compounds, and their mixture are indicated in Figure 3A. By being divided by the standard spectrum ($A_{N,\lambda i\;}=\beta_{\lambda i}\;C_{N}^{o}$) of one of the compounds in the binary mixture, Equation (21) becomes

(24) $$\frac{{\text{A}}_{\text{m},\lambda i\;}}{\beta_{\lambda i}\;C_{N}{}^{o}}=\;\frac{\alpha_{\lambda i}\;{\text{C}}_{\text{M}}}{\beta_{\lambda i}\;{\text{C}}_{\text{N}}{}^{\text{o}}}+\;\frac{\beta_{\lambda i}\;{\text{C}}_{\text{N}}}{\beta_{\lambda i}\;C_{N}{}^{o}}$$

Figure 3B shows the ratio spectra of analytes and their binary mixture. If CWT is applied to Equation (24), the following equation can be obtained

(25) $$CWT\left[\frac{A_{m,\lambda i\;}}{\beta_{\lambda i}\;C_{N}^{o}}\right]=CWT\;\left[\frac{\alpha_{\lambda i}\;}{\beta_{\lambda i}\;}\right]\frac{\;C_{M\;}}{\;C_{N}^{o}}+CWT\left[\frac{\beta_{\lambda i}\;}{\beta_{\lambda i}\;}\;\right]\frac{\;C_{N\;}}{\;C_{N}^{o}}$$

If $CWT\left[\frac{\beta_{\lambda i}\;}{\beta_{\lambda i}\;}\;\right]\frac{\;C_{N\;}}{\;C_{N}^{o}}$ = 0, then we obtain

(26) $$\text{CWT}[\frac{{\text{A}}_{\text{m},\text{λi }}}{\beta_{\lambda i}\;{\text{C}}_{\text{N}}^{\text{o}}}]=\text{CWT}\;[\frac{\alpha_{\lambda \text{i}}\;}{\beta_{\lambda i}\;}]\frac{\;{\text{C}}_{\text{M }}}{\;{\text{C}}_{\text{N}}^{\text{o}}}$$

The ratio-CWT amplitudes of the binary mixture given in Equation (26) depend only on C_M_ and ${{\text{C}}_{\text{N}}}^{\text{o}}$ regardless of C_N_ (e.g., see Figure 3C).

## Ratio spectra-continuous wavelet transform-zero crossing

In RS-CWT-ZC approach (Dinç et al., 2005a), if a mixture of three analytes (X, Y, and Z) is considered and the absorbance of this ternary mixture is measured at λ_i_, the following mathematical expression (Charlotte Grinter and Threlfall, 1992) would be given

(27) $${\text{A}}_{mix,\;\lambda i}\;=\;\alpha_{X,\;\lambda i}{\text{C}}_{\text{X}}+\;\beta_{Y,\;\lambda i}{\text{C}}_{\text{Y}}+\;\gamma_{Z,\;\lambda i}{\text{C}}_{\text{Z}}$$

Where A_*mix*, λ*i*_ is the absorbance of the ternary mixture at wavelength λ_*i*_, and coefficients α_*X*, λ*i*_, β_*Y*, λ*i*_, and γ_*Z*, λ*i*_ denote the absorptivities of X, Y, and Z, respectively. C_X_, C_Y_, and C_Z_ represent the concentrations of X, Y, and Z, respectively.

If Equation (27) is divided by the spectrum of a standard solution (${\text{C}}_{\text{X}}^{\text{o}}$) of one of the compounds in the ternary mixture, we have the following equation:

(28) $$\frac{\;{\text{A}}_{mix,\;\lambda i\;}}{\alpha_{X,\;\lambda i}{{\text{C}}^{o}}_{\text{X}}}\;=\;\frac{\;\alpha_{X,\;\lambda i}{\text{C}}_{\text{X}}\;}{\alpha_{X,\;\lambda i}{{\text{C}}^{o}}_{\text{X}}}+\;\frac{\;\beta_{Y,\;\lambda i}{\text{C}}_{\text{Y }}}{\alpha_{X,\;\lambda i}{{\text{C}}^{o}}_{\text{X}}}+\;\frac{\;\gamma_{Z,\;\lambda i}{\text{C}}_{\text{Z }}}{\alpha_{X,\;\lambda i}{\text{C}}_{\text{X}}^{\text{o}}}$$

If CWT is applied to Equation (28), the following equation can be obtained

(29) $$\text{CWT}\left[\frac{\;{\text{A}}_{mix,\;\lambda i\;}}{\alpha_{X,\;\lambda i}{{\text{C}}^{\text{o}}}_{\text{X}}}\right]=\text{ CWT}\left[\frac{\;\beta_{\text{Y},\;\lambda i}{\text{C}}_{\text{Y }}}{\alpha_{\text{X},\;\lambda i}{{\text{C}}^{\text{o}}}_{\text{X}}}\right]+\text{CWT}\left[\frac{\;\gamma_{\text{Z},\;\lambda i}{\text{C}}_{\text{Z }}}{\alpha_{\text{X},\;\lambda i}{{\text{C}}^{\text{o}}}_{\text{X}}}\right]$$

Equation (29) indicates that the CWT amplitudes of the ratio spectra of the ternary mixture are dependent only on C_Z_ and ${{\text{C}}_{\text{X}}}^{\text{o}}$ regardless of the concentrations of other compounds.

## Double divisor ratio spectra-continuous wavelet transform

In addition to RS-CWT-ZC approach, the spectral resolution of ternary mixtures could be effectively done by DDRS-CWT approach (Dinç and Baleanu, 2008a) as follows.

When two compounds in the ternary mixture is used as a *double divisor*, we have

(30) $${\text{A}}_{\text{mix},\;\lambda \text{i}}^{\text{o}}=\;\alpha_{\text{X},\;\lambda \text{i}}{\text{C}}_{\text{X}}^{\text{o}}\text{+ }\beta_{\text{Y},\;\lambda \text{i}}\;{\text{C}}_{\text{Y}}^{\text{o}}$$

By dividing Equation (27) and (30), we obtain as follows

(31) $$\begin{array}{l} \frac{{\text{A}}_{\text{mix},\;\lambda \text{i}}}{\alpha_{\text{X},\;\lambda \text{i}}{\text{C}}_{\text{X}}^{\text{o}}\text{+ }\beta_{\text{Y},\;\lambda \text{i}}\;{\text{C}}_{\text{Y}}^{\text{o}}}=\frac{\alpha_{\text{X},\;\lambda \text{i}}{\text{C}}_{\text{X}}}{\alpha_{\text{X},\;\lambda \text{i}}{\text{C}}_{\text{X}}^{\text{o}}\text{+ }\beta_{\text{Y},\;\lambda \text{i}}\;{\text{C}}_{\text{Y}}^{\text{o}}}\\ +\frac{\beta_{\text{Y},\;\lambda \text{i}}{\text{C}}_{\text{Y}}}{\alpha_{\text{X},\;\lambda \text{i}}{\text{C}}_{\text{X}}^{\text{o}}\text{+ }\beta_{\text{Y},\;\lambda \text{i}}\;{\text{C}}_{\text{Y}}^{\text{o}}}+\frac{\gamma_{\text{Z},\;\lambda \text{i}}{\text{C}}_{\text{Z}}}{\alpha_{\text{X},\;\lambda \text{i}}{\text{C}}_{\text{X}}^{\text{o}}\text{+ }\beta_{\text{Y},\;\lambda \text{i}}\;{\text{C}}_{\text{Y}}^{\text{o}}} \end{array}$$

Equation (31) can be simplified to

(32) $$\frac{{\text{A}}_{\text{mix},\;\lambda \text{i}}}{\alpha_{X,\;\lambda i}{\text{C}}_{\text{X}}{}^{\text{o}}+\;\beta_{\text{Y},\;\lambda i}\;{\text{C}}_{\text{Y }}{}^{\text{o}}}=\text{ k}+\;\frac{\;\gamma_{\text{Z},\;\lambda \text{i}}{\text{C}}_{\text{Z}}}{\;\alpha_{\text{X},\;\lambda \text{i}}{\text{C}}_{\text{X}}{}^{\text{o}}\text{+ }\beta_{\text{Y},\;\lambda \text{i}}\;{\text{C}}_{\text{Y }}{}^{\text{o}}}$$

Where $k=\frac{{\text{α}}_{\text{X},\text{ λi}}C_{X}+\;\beta_{Y,\;\lambda i}C_{Y}}{{\text{α}}_{\text{X},\text{ λi}}C_{X}^{o}+\;\beta_{Y,\;\lambda i}\;C_{Y}^{o}}$ represents a constant for a given concentration range with respect to λ_i_ in a certain region or point of wavelength.

A typical case is when ${{\text{C}}_{\text{X}}}^{\text{o}}$ and ${{\text{C}}_{\text{Y}}}^{\text{o}}$ are the same or very close to each other, namely ${{\text{C}}_{\text{X}}}^{\text{o}}$ = ${{\text{C}}_{\text{Y}}}^{\text{o}}$ or $≅{{\text{C}}_{\text{X}}}^{\text{o}}≅{{\text{C}}_{\text{Y}}}^{\text{o}}$. Therefore, we obtain

(33) $$\alpha_{\text{X},\;\lambda \text{i}}{\text{C}}_{\text{X}}^{\text{o}}\text{+ }\beta_{\text{Y},\;\lambda \text{i}}\;{\text{C}}_{\text{Y}}^{\text{o}}=\;{\text{C}}_{\text{X}}^{\text{o}}\;(\alpha_{\text{X},\;\lambda \text{i}}\text{+}\beta_{\text{Y},\;\lambda \text{i}})$$

and Equation (32) can be written as

(34) $$\frac{{\text{A}}_{\text{mix},\;\lambda \text{i}}}{\;\alpha_{\text{X},\;\lambda \text{i}}{\text{C}}_{\text{X}}^{\text{o}}\text{+ }\beta_{\text{Y},\;\lambda \text{i}}\;{\text{C}}_{\text{Y }}^{\text{o}}}=\;k+\;\frac{\;\gamma_{\text{Z},\;\lambda \text{i}}{\text{C}}_{\text{Z}}}{\;{\text{C}}_{\text{X}}^{\text{o}}\;\left(\;\alpha_{\text{X},\;\lambda \text{i}}\text{+ }\beta_{\text{Y},\;\lambda \text{i}}\right)}$$

After applying CWT to Equation (31), we have

(35) $${\text{CWT}}_{\left(a,b\right)}\left(\frac{{\text{A}}_{\text{mix},\;\lambda \text{i}}}{\;\alpha_{\text{X},\;\lambda \text{i}}\text{+ }\beta_{\text{Y},\;\lambda \text{i}}\;}\right)\frac{\text{1}}{{\text{C}}_{\text{X}}^{\text{o}}}={\text{CWT}}_{\left(a,b\right)}\left(\frac{\gamma_{\text{Z},\;\lambda \text{i}}{\text{C}}_{\text{Z}}}{\left(\alpha_{\text{X},\;\lambda \text{i}}\text{+ }\beta_{\text{Y},\;\lambda \text{i}}\right)}\right)\frac{\text{1}}{{\text{C}}_{\text{X}}^{\text{o}}}$$

or

(36) $${\text{CWT}}_{\left(a,b\right)}\left(\frac{{\text{A}}_{\text{mix},\;\lambda \text{i}}}{\;\alpha_{\text{X},\;\lambda \text{i}}\text{+ }\beta_{\text{Y},\;\lambda \text{i}}\;}\right){\text{=CWT}}_{\left(a,b\right)}\left(\frac{\gamma_{\text{Z},\;\lambda \text{i}}}{\left(\;\alpha_{\text{X},\;\lambda \text{i}}\text{+ }\phi_{\text{Y},\;\lambda \text{i}}\right)}\right){\text{C}}_{\text{Z}}$$

In Equation (36), C_Z_ is to proportional to the coefficients, ${\text{CWT}}_{\left(\text{a},\text{b}\right)}\left(\frac{{\text{A}}_{\text{m}}\text{ix},\text{ λi}}{{\;\alpha }_{\text{X},\text{ λi}}+\;\beta_{\text{Y},\;\lambda i}\;}\right)$, at λi. If this procedure is separately applied for pure Z and its ternary mixture, the CWT_(a, b)_ coefficients are coincided at some characteristic point or region of wavelength, independent upon both C_X_ and C_Y_.

## Wavelet transform-based UV spectroscopic analysis of pharmaceuticals

Typical applications of CWT and FWT algorithms for UV spectroscopic analysis of pharmaceuticals are displayed in Tables 2–5. It is worth mentioning that WT could be solely applied to raw spectra and ratio spectra (as above-specified) as well as utilized as a hybrid approach (FWT-derivative, FWT-CWT-zero crossing, WT combined with multivariate calibration) for the simultaneous determination of analytes in pharmaceutical binary and ternary mixtures. It was shown that wavelet analysis of UV spectroscopic data was performed by using Wavelet Toolbox and m-file in MATLAB software. The numerous works provided by Dinç and co-workers have clearly highlighted the success of WT-based UV spectroscopic analysis for multicomponent synthetic mixtures, veterinary and pharmaceutical dosage forms as well as different types of test (e.g., assay, *in vitro* dissolution, stability indicating). Most studies proved it to be suitable for the routine analysis of dosage forms with good precision and accuracy, comparable to HPLC.

**Table 2:** Applications of the continuous wavelet transform-zero crossing technique to UV spectroscopic analysis of pharmaceuticals.

**Pharmaceuticals**	**Method**	**Wavelet Families**	**Type of data**	**References**
Thiamine HCl, pyridoxine HCl	CWT-zero crossing	Daubechies, Biorthogonal	UV absorption spectra	Dinç and Baleanu, 2003a
Hydrochlorothiazide, spironolactone	CWT-zero crossing	Daubechies, Biorthogonal	UV absorption spectra	Dinç et al., 2003
Thiamine HCl; pyridoxine HCl	CWT-zero crossing	Mexican hat function, Meyer	UV absorption spectra	Dinç and Baleanu, 2003b
Thiamine HCl, pyridoxine HCl	CWT-zero crossing	Gaussian1, Gaussian2	UV absorption spectra	Dinç and Baleanu, 2004a
Caffeine, propyphenazone	DWT-CWT-zero crossing	Mexican and Haar	UV absorption spectra	Dinç et al., 2004a
Benazepril, hydrochlorothiazide	DWT-CWT-zero crossing	Coiflets2 and Gaussian2	UV absorption spectra	Dinç and Baleanu, 2004b
Hydrochlorothiazide, Spironolactone	CWT-zero crossing	Haar, Mexican hat function	UV absorption spectra	Dinç et al., 2004c
Benazepril, hydrochlorothiazide	CWT-zero crossing	Mexican, Haar, Daubechies3	UV absorption spectra	Dinç and Baleanu, 2004c
Ascorbic acid, acetylsalicylic acid	CWT-zero crossing	Mexican hat function	UV absorption spectra	Dinç et al., 2005b
Diminazene aceturate and phenazone	CWT-zero crossing	Reverse Biorthogonal	UV absorption spectra	Dinç et al., 2005c
Quinapril, hydrochlorothiazide	CWT-zero crossing	Mexican hat wavelet function	UV absorption spectra	Dinç and Baleanu, 2007a
Oxfendazole and oxyclozanide	CWT-zero crossing	Mexican hat function	UV absorption spectra	Dinç and Baleanu, 2007c
Levodopa, benserazide	CWT-zero crossing	Symlets	UV absorption spectra	Dinç et al., 2007d
Chlortetracycline, benzocaine	CWT-zero crossing	Coiflets	UV absorption spectra	Dinç et al., 2007c
Pyridoxine hydrochloride, isoniazide	CWT-zero crossing	Mexican hat function	UV absorption spectra	Üstündag et al., 2008
Risedronate sodium	CWT-zero crossing	Morlet, Biorthogonal	UV absorption spectra	Ugurlu et al., 2008
ampicillin sodium, sulbactam sodium	CWT-zero crossing	Mexican hat function, Symtles	UV absorption spectra	Dinç and Baleanu, 2009a
Paracetamol, chloroxozone	CWT-zero crossing	Mexican hat function, Daubechies, Symplets, Coiflets, Biortogonal, Gaussian	UV absorption spectra	Dinç et al., 2009a
Levamisole, triclabendazole	CWT-zero crossing	Biorthogonal	UV absorption spectra	Dinç et al., 2009b
Telmisartan, hydrochlorothiazide	CWT-zero crossing	Gaussian, Biorthogonal	UV absorption spectra	Dinç and Baleanu, 2009b
Perindopril, indapamide	CWT-zero crossing	Haar and Biorthogonal1.5	UV absorption spectra	Pektaş et al., 2009
Valsartan, amlodipine	CWT-zero crossing	Daubechies, Dmeyer	UV absorption spectra	Dinç and Baleanu, 2010a
Metformin hydrochloride, glibenclamide	DWT-CWT-zero crossing	Daubechies, Reverse Biorthogonal, Gaussian	UV absorption spectra	Sohrabi et al., 2011
Trimethoprim, sulphamethoxazole	CWT-zero crossing	Biorthogonal, Coiflets, Daubechies, Haar	UV absorption spectra	Dinç et al., 2011b
Amlodipine, atorvastatine	CWT-zero crossing	Mexican hat function	UV absorption spectra	Shariati-Rad et al., 2012
Estradiol valerate, cyproterone acetate	CWT-zero crossing	Symlets	UV absorption spectra	Dinç et al., 2013a
Lamivudine, zidovudine	CWT-zero crossing	Mexican hat wavelet, Symlets, Daubechies	UV absorption spectra	Dinç et al., 2013b
Diphenhydramine hydrochloride	CWT-zero crossing	Biorthogonal	UV absorption spectra	Devrim et al., 2014
Ambroxol hydrochloride, doxycycline	CWT-zero crossing	Haar wavelet function	UV absorption spectra	Darwish et al., 2014
Oxfendazole, oxyclozanide	MOFrFT-CWT-zero-crossing	Mexican hat	UV absorption spectra	Dinç et al., 2015
Atenolol, chlorthalidone	CWT-zero crossing	Coiflet, Mexican Hat function	UV absorption spectra	Dinç et al., 2017b
Valsartan, hydrochlorothiazide	CWT-zero crossing	Mexican hat function, Daubechies	UV absorption spectra	Dinç et al., 2017a

**Table 3:** Applications of the wavelet transform-multivariate approaches to UV spectroscopic analysis of pharmaceuticals.

**Pharmaceuticals**	**Method**	**Families**	**Type of data**	**References**
Tetramethrin, propoxur; piperonil butoxide	CWT-PCR, CWT-PLS	Mexican hat function	UV absorption spectra	Dinç et al., 2004b
Paracetamol, ascorbic acid, acetylsalicylic acid	DWT-CLS, DWT-PLS	Haar	UV absorption spectra	Dinç et al., 2006a

**Table 4:** Applications of the ratio spectra-continuous wavelet transform, ratio spectra- continuous wavelet transform-zero crossing approaches to UV spectroscopic analysis of pharmaceuticals.

**Pharmaceuticals**	**Method**	**Families**	**Type of data**	**References**
Paracetamol, acetylsalicylic acid, caffeine	Ratio spectra-CWT-ZC	Mexican hat function	UV ratio spectra	Dinç et al., 2005a
Diminazene aceturate and phenazone	Ratio spectra-CWT	Reverse Biorthogonal	UV ratio spectra	Dinç et al., 2005c
Paracetamol, metamizol, caffeine	Ratio spectra-CWT-ZC	Mexican hat function, Reverse biorthogonal, Biorthogonal	UV ratio spectra	Dinç et al., 2006b
Levamizol, oxycloanide	Ratio spectra-CWT	Daubechies	UV ratio spectra	Dinç et al., 2007a
oxfendazole and oxyclozanide	Ratio spectra-CWT	Morlet	UV ratio spectra	Dinç and Baleanu, 2007c
Ascorbic acid, acetylsalicylic acid and paracetamol	Double divisor-ratio spectra-CWT	Haar, Mexican hat function	UV- double divisor-ratio spectra	Dinç and Baleanu, 2008a
vitamin C, aspirin	Ratio spectra-CWT	Biorthogonal	UV ratio spectra	Dinç and Baleanu, 2008b
valsartan and hydrochlorothiazide	Ratio spectra-CWT	Mexican hat function, Coiflets	UV ratio spectra	Dinç et al., 2017a

**Table 5:** Applications of the fractional wavelet transform and its combination with other chemometric techniques to UV spectroscopic analysis of pharmaceuticals.

**Pharmaceuticals**	**Method**	**Families**	**Type of data**	**References**
Ampicillin, sulbactam	FWT-derivative method	–	UV absorption data	Dinç and Baleanu, 2006
Lacidipine and its photodegradation product	FWT-CWT	Mexican hat function	UV absorption data	Dinç et al., 2006c
Cilazapril, hydrochlorothiazide	FWT-PLS	–	UV absorption data	Dinç et al., 2007b
Paracetamol, propiphenazone, caffeine and thiamine	FWT-PCR, FWT-PLS, FWT-ANN	–	UV absorption data	Dinç et al., 2008
Amlodipine, valsartan	FWT-PLS1, FWT-PLS2	–	UV absorption data	Çelebier et al., 2010
Trimethoprim, sulfachloropyridazine sodium	FWT-derivative method	–	UV absorption data	Kanbur et al., 2010
Atorvastatin, amlodipine	FWT-CWT	Mexican wavelet hat function	UV absorption data	Dinç and Baleanu, 2010b
Trimethoprim, sulphamethoxazole	FWT-PCR, FWT-PLS	–	UV absorption data	Dinç et al., 2010
of oxytetracycline and flunixin megluminein	FWT-PCR, FWT-PLS	–	UV absorption data	Kambur et al., 2011
Olmesartan modoxomil, hydrochlorothiazide	FWT-CWT	Mexican wavelet hat function	UV absorption data	Dinç et al., 2011a
Thiamine HCl, pyridoxine HCl, lidocaine HCl	FWT-PCR, FWT-PLS,	–	UV absorption data	Dinç and Baleanu, 2012
	FWT-CWT-PCR, FWT-CWT-PLS			
Melatonin and its photodegradation	FWT-CWT	Biorthogonal, symplets	UV absorption data	Dinç et al., 2012

## Conclusions

In the point of view of UV spectroscopic analysis of multicomponent mixtures, CWT-based UV spectroscopic methods have outperformed both conventional and derivative UV spectroscopy in resolving spectrally binary and ternary mixtures. Nevertheless, wavelet analysis may not also have a sufficient power to resolve overlapping spectra of analytes in samples due to similarity of molecular structures and signal frequencies in some cases. They may not give desirable results for a complex mixture containing more than three compounds and/or a significant difference in ratios of active ingredients. In such a case, the use of WT coupled with chemometric PLS and PCR calibrations is advisable. Undoubtedly, however, wavelets can still be used as a mathematical prism for signal analysis because they can offer many possibilities such as baseline correction, noise removal and resolution of overlapping peaks, when the frequencies of analyzed components are significantly different from each other.

## Author contributions

Contributions of ED are planning and writing of the review paper. Contributions of ZY are literature review, collection, editing, and format arrangement.

### Conflict of interest statement

The authors declare that the research was conducted in the absence of any commercial or financial relationships that could be construed as a potential conflict of interest.
